# A kinetic assay of total lipase activity for detecting lysosomal acid lipase deficiency (LAL‐D) and the molecular characterization of 18 LAL‐D patients from Russia

**DOI:** 10.1002/jmd2.12050

**Published:** 2019-06-03

**Authors:** Nikolay Mayanskiy, Ekaterina Brzhozovskaya, Alexander Pushkov, Tatiana Strokova, Nikolay Vlasov, Andrej Surkov, Olga Gundobina, Kirill Savostianov

**Affiliations:** ^1^ Russian Children Clinical Hospital Pirogov Russian National Research Medical University Moscow Russia; ^2^ Laboratory department National Medical Research Center for Children's Health Moscow Russia; ^3^ Laboratory for molecular genetics and cellular biology National Medical Research Center for Children's Health Moscow Russia; ^4^ Department for pediatric gastroenterology, hepatology and nutrition Federal Research Center for Nutrition and Biotechnology Moscow Russia; ^5^ Department for pediatrics Filatov's Children Hospital, No.5 Saint‐Petersburg Russia; ^6^ Gastroenterology Chair State Pediatric Medical University Saint‐Petersburg Russia; ^7^ Department for gastroenterology National Medical Research Center for Children's Health Moscow Russia

**Keywords:** cholesterol ester storage disease, Lalistat‐2‐independent assay, lysosomal acid lipase, Wolman disease

## Abstract

Laboratory diagnostics of lysosomal acid lipase deficiency (LAL‐D), a rare disorder associated with *LIPA* alterations, are based on the evaluation of LAL activity. In dry blood spots (DBS) submitted for LAL‐D diagnostics (the screening cohort) over a two‐year period or obtained from a cohort of retrospective LAL‐D patients, we measured: (1) LAL activity using a two‐reaction assay with 4‐methylumbelliferone palmitate (4‐MU‐Palm) and Lalistat‐2, a specific LAL inactivator; (2) total lipase (TL) activity by a 1‐hour kinetic 4‐MU‐Palm cleavage reaction (no Lalistat‐2). The TL activity was expressed as the area under the kinetic curve after 1 hour (TL‐AUC_1h_) of the reaction and presented as the median (min‐max). LAL activity was reduced in 30/537 individuals from the screening cohort, among which *LIPA* sequencing revealed six patients and one carrier. Overall, 16 (89%) individuals among six novel and 12 retrospective LAL‐D patients carried at least one c.894G>A mutation (six were homozygous). The TL‐AUC1h in nonLAL‐D specimens with normal LAL activity (n = 90) was unambiguously higher (9471 [4015‐23 585] RFU*h/punch) compared to LAL‐D patients, including six new and nine retrospective patients (1810 [357‐2608] RFU*h/punch). Importantly, in 13/15 examined nonLAL‐D specimens with reduced LAL activity the TL‐AUC1h was above a threshold of 2652 RFU*h/punch. Applying this threshold, the TL‐AUC1h index discriminated all LAL‐D patients (100% sensitivity) and 103/105 nonLAL‐D specimens (98% specificity). Given that there is no need for Lalistat‐2 and two parallel enzymatic reactions in conjunction with high sensitivity and specificity, the kinetic assay seems to be practical for LAL‐D screening.

**Synopsis:**

Lysosomal acid lipase deficiency responsible for Wolman disease and cholesterol ester storage disease could be reliably detected using a kinetic assay of total lipase activity with a fluorogenic substrate.

## 1 INTRODUCTION

Lysosomal acid lipase deficiency (LAL‐D) is an autosomal recessive disorder that relates to a mutation of the *LIPA* gene. LAL is an acid hydrolase of cholesteryl esters; thus, body lipid metabolism disturbances are characteristic of this disease.^1^ Infantile‐onset LAL‐D, known as Wolman disease, is the more severe and rare form of LAL‐D, while another form, cholesteryl ester storage disease (CESD), may begin in childhood and adulthood. The real incidence of LAL‐D is not known, but estimates are 1:40000 to 1:300000 depending on the geographical region and ethnic background.^1^, ^2^, ^3^ There have been no comprehensive reports regarding LAL‐D in Russia in the international literature so far.

LAL‐D diagnostics are based on detecting reduced enzymatic activity toward a lipase substrate supported by *LIPA* sequencing.^1^, ^4^ Although no specific substrate for LAL is commercially available so far, the use of the fluorogenic 4‐methylumbelliferone palmitate ester (4‐MU‐Palm) has been shown to be useful for LAL‐D diagnostics.^5^ For overcoming potential interference of other lipase(s) in the 4‐MU‐Palm cleavage, the use of the specific LAL inhibitor, Lalistat‐2, has been proposed by Hamilton et al.^6^, ^7^ In this assay, LAL activity is measured as the difference between two activity values obtained without Lalistat‐2 and in the presence of this substance. The Lalistat‐2‐dependent LAL activity method is now widely used for LAL‐D diagnostics as well as for other research purposes,^8^, ^9^, ^10^ although the total error of a two‐reaction assay could be intrinsically high. In a recent study by Gelb et al, a new, promisingly specific substrate for LAL has been discovered, which was suitable for fluorimetric as well as mass spectrometry assays^11^; however, this substrate is not practically available yet.

With the advent of enzyme replacement therapy by a recombinant human LAL, sebelipase alfa, LAL‐D has become a potentially treatable disease. A number of clinical trials have demonstrated amelioration of clinical and laboratory manifestations of LAL‐D with increased life expectancy.^12^ Thus, an accurate diagnosis is important for a timely initiation of LAL‐D treatment.

Herein, we report the two‐year experience of performing the Lalistat‐2‐dependent LAL activity assay for LAL‐D diagnostics in a cohort of individuals screened clinically for LAL‐D signs and describe a kinetic, Lalistat‐2‐independent modification of the conventional LAL activity assay and evaluate its suitability for LAL‐D diagnostics. Additionally, we provide the description of *LIPA* alterations found in 18 Russian LAL‐D patients.

## 2 MATERIALS AND METHODS

All described laboratory procedures were performed at the Laboratory Department, National Medical Research Center for Children's Health, Moscow.

### 2.1 Implementation study for the Lalistat‐2‐dependent LAL activity assay

For the implementation of the Lalistat‐2‐dependent LAL activity assay,^6^ DBS were obtained from healthy volunteers among the hospital staff as well as by using redundant blood specimens from children undergoing elective surgery, without known chronic conditions, which were submitted to the core lab for routine complete blood count analysis. In all these specimens, white blood cell (WBC) and platelet (PLT) counts were measured using a hemocytometer; only specimens with values within the reference interval were used.

### 2.2 LAL‐D screening cohort

This cohort included DBS obtained from individuals, who were suspected of having LAL‐D after clinical screening (the presence of unexplained hepato‐ and/or splenomegaly, characterized by an increase in transaminase activity by ≥1.5 times the upper reference limit, with or without a disturbed serum lipid profile), and submitted to our laboratory for the measurement of LAL activity between June 2016 and July 2018.

### 2.3 Retrospective LAL‐D patients

This cohort included LAL‐D affected individuals, who were either diagnosed at or attending our institutions, including the National Medical Research Center for Children's Health (Moscow), the Federal Research Center for Nutrition and Biotechnology (Moscow), and Filatov's Children Hospital No.5 (St. Petersburg), starting in 2012. DBS for the present analyses were obtained during a LAL‐D diagnostic workflow or routine patient checkup.

### 2.4 DBS specimen preparation

For all analyses presented in this study, DBS were prepared by spotting EDTA venous blood on filter paper (Whatman 903). Fresh DBS were analyzed within three days upon preparation and were archived at −25°C in individual plastic bags with a desiccant pouch for further storage.

### 2.5 Lalistat‐2‐dependent LAL activity assay

This assay was carried out essentially as described by Hamilton et al^6^ using 4‐MU‐Palm as a substrate and the LAL inhibitor, Lalistat‐2. Briefly, 40 μL aliquots of DBS water extracts were preincubated with 10 μL of 30 μM Lalistat‐2 or water for 10 minutes in a 96‐well flat bottom black plate. Next, 150 μL of reaction buffer solution containing 4‐MU‐Palm (0.35 mM) and cardiolipin (0.032%) in a 0.15 M acetate buffer with 1% Triton X‐100, pH 4.0 was added. The plate was sealed and incubated at 37°C for 2.5 hours. Following the incubation, eight 4‐MU standards serially diluted in 200 μL of water (range, 0‐2.5 nmol 4‐MU) were added to the plate. A hundred μL of water was added to all wells, and the fluorescence was measured in an Infinite 200 M plate reader (Tecan, Austria) (excitation λ = 330 nm; emission λ = 460 nm). LAL activity was calculated by subtracting the value in the inhibited reaction (with Lalistat‐2) from the value of reaction without the inhibitor; the latter was considered the total lipase activity. The limit of detection was 0.001 nmol 4‐MU/punch/1 hour.

### 2.6 Kinetic measurement of total lipase activity

For the kinetic assay, 40 μL of DBS water extracts and 150 μL of the reaction buffer described above were mixed in a 96‐well, flat bottom black plate. The plate was immediately placed into the Infinite 200 M plate reader, which was prewarmed to 37°C, and the kinetic measurement was started. Changes in fluorescence generated by the release of 4‐MU upon the lipase‐mediated 4‐MU‐Palm cleavage were recorded at 5‐minutes intervals. Initially, to keep the assay time in accordance with the Hamilton method,^6^ we performed kinetic measurements for 150 minutes, that is, for a total of 31 kinetic cycles. However, a 1‐hour kinetic measurement (13 kinetic cycles) was sufficient (not shown), and we used this time period for further kinetic analyses. As a kinetic measure of the total lipase activity, we chose the area under the kinetic curve after 1 hour (AUC_1h_) of the 4‐MU‐Palm cleavage reaction. Total lipase AUC_1h_ (TL‐AUC_1h_) was calculated with Magellan software (version 7.2; Tecan) and expressed as relative fluorescence units (RFU)*1 hour per punch. All TL‐AUC_1h_ values were corrected for the time point of 0 minute by subtracting the RFU value of the 1st measurement from the other measurements in the same specimen. All kinetic assays were performed in duplicate.

### 2.7 LIPA gene analysis

Genomic DNA was extracted from DBS using the QuickGene DNA Tissue Kit S (AutoGen, Holliston, Massachusetts). All exonic regions along with the flanking intronic regions of *LIPA* were sequenced on an ABI 3500 DNA Sequencer (Applied Biosystems). Nucleotide sequences were analyzed using the Sequencing Analysis software version 5.4 (Applied Biosystems). Alamut 3.2 software (Interactive Biosoftware, Rouen, France) was used for the alignment analysis and mutation identification.

### 2.8 Statistics

Statistical analysis was performed using IBM SPSS Statistics for Windows, version 21.0 (IBM Corp., Armonk, New York). Median values were compared using the Mann‐Whitney *U* test; correlation between LAL activity and age was assessed by means of the Spearman's *r* coefficient. The tests were considered statistically significant at *P* < .05.

## 3 RESULTS

### 3.1 The implementation of the Lalistat‐2‐dependent LAL assay and definition of a lower limit boundary for LAL activity

In 2016, we implemented the LAL assay described by Hamilton et al^6^ locally. The implementation study included DBS obtained from 43 healthy individuals and seven patients with previously confirmed diagnosis of LAL‐D. In healthy individuals, LAL activity varied from 0.070 to 0.549 nmol 4‐MU/punch/1 hour, with a median of 0.252 nmol 4‐MU/punch/1 hour. The LAL‐D patients demonstrated a range of LAL activity from < 0.001 to 0.052 nmol 4‐MU/punch/1 hour (median, 0.010 nm/punch). Using the robust method of calculating the reference interval (Clinical and Laboratory Standards Institute [CLSI]. Defining, Establishing, and Verifying Reference Intervals in the Clinical Laboratory; Approved Guideline, Third Edition CLSI document C28‐A3, 2008), the lower limit of LAL activity was defined at 0.07 nmol 4‐MU/punch/1 hour, comprising 28% of the median LAL activity in the healthy group. LAL activity < 0.07 nmol 4‐MU/punch/1 hour was classified as low.

### 3.2 Examination of LAL activity in the LAL‐D screening cohort

Using the implemented Lalistat‐2‐dependent LAL activity assay, we analyzed 537 dB specimens submitted from 57 cities from all over Russia between June 2016 and July 2018. The specimens were obtained from individuals of variable age, ranging from ≤1 months to 66 years; 239 (44.5%) specimens were from children ≤ 5 years of age. In 30 (5.6%) of these 537 dB, LAL activity was below the defined lower reference boundary of 0.07 nmol 4‐MU/punch/1 hour. *LIPA* sequencing was performed in all 30 specimens and revealed six individuals with pathogenic mutations affecting both *LIPA* alleles and one individual carrying a pathogenic mutation in one *LIPA* allele with an intact second allele. In the remaining 23 individuals with reduced LAL activity, no significant *LIPA* sequence alterations were detected. Thus, six new genetically confirmed LAL‐D cases were detected in the examined cohort. Detailed characteristics of the affected individuals are provided below.

Among 530 nonaffected individuals, LAL activity varied from 0.010 to 2.8 nmol 4‐MU/punch/1 hour, with a median of 0.257 nmol 4‐MU/punch/1 hour (Table 1, “No LAL‐D, all” raw). LAL activity had a strong reverse correlation with age (*r* = −.371, *P* < .001) and was significantly increased in children ≤ 5 years of age (Table 1), decreasing afterwards and reaching adult values by the age of 10‐15 years (not shown). The rate of specimens with LAL activity < 0.07 nmol 4‐MU/punch/1 hour was 4.3% (23/530).

**Table 1:** LAL activity in DBS submitted for LAL‐D screening by the Lalistat‐2‐dependent assay

Group (No.)	LAL activity, nmol 4‐MU/punch/ 1 h		No. (%) of specimens with LAL activity < 0.07 nmol 4‐MU/punch/1 h^a^
	Median (P25; P75)	Range	
No LAL‐D, all (530)	0.257 (0.162; 0.414)	0.010‐2.8	23 (4.3%)
≤5 years old (238)	0.335 (0.236; 0.480)^b^	0.022‐2.8	8 (3.4%)
>5 years old (292)	0.216 (0.130; 0.329)	0.010‐1.5	15 (5.1%)
Carrier, new case (1)^c^	0.030	‐	1/1
LAL‐D, new cases (6)	0.012 (0.006; 0.052)	< 0.001‐0.060	6 (100%)

Abbreviations: DBS, dry blood spots; LAL, lysosomal acid lipase; LAL‐D, lysosomal acid lipase deficiency.

a The lower reference boundary for LAL activity defined locally.

b *P* < .001 vs > 5 years old.

c c.796G>T *LIPA* heterozygote.

### 3.3 Kinetic assay of total lipase activity in LAL‐D patients and nonaffected individuals

While implementing the Lalistat‐2‐dependent LAL assay in our laboratory, we found that the total lipase activity (ie, values from the reaction of 4‐MU‐Palm‐cleavage without Lalistat‐2) in healthy individuals and LAL‐D patients did not intersect (minimum in the healthy group of 0.217 vs maximum in the LAL‐D group of 0.126 nmol 4‐MU/punch/1 hour). The addition of Lalistat‐2 inhibited 62% of the total lipase activity in specimens from healthy individuals, leaving 38% attributed to nonLAL lipases. In LAL‐D specimens lacking LAL, Lalistat‐2 suppressed <1% of the total lipase activity, implying that the cleavage of 4‐MU‐Palm in LAL‐D specimens was mediated almost exclusively by nonLAL lipases, upon which Lalistat‐2 had no effect. Interestingly, absolute values of nonLAL lipase activity in healthy individuals were nearly 20% higher than those in LAL‐D patients (median values of 0.141 vs 0.110 nmol 4‐MU/punch/1 hour, respectively; *P* < .001). This finding suggested that, in addition to LAL, the activity of other enzyme(s) responsible for 4‐MU‐Palm cleavage could be suppressed in LAL‐D patients, thus reducing the total lipase activity.

**Figure 1:**
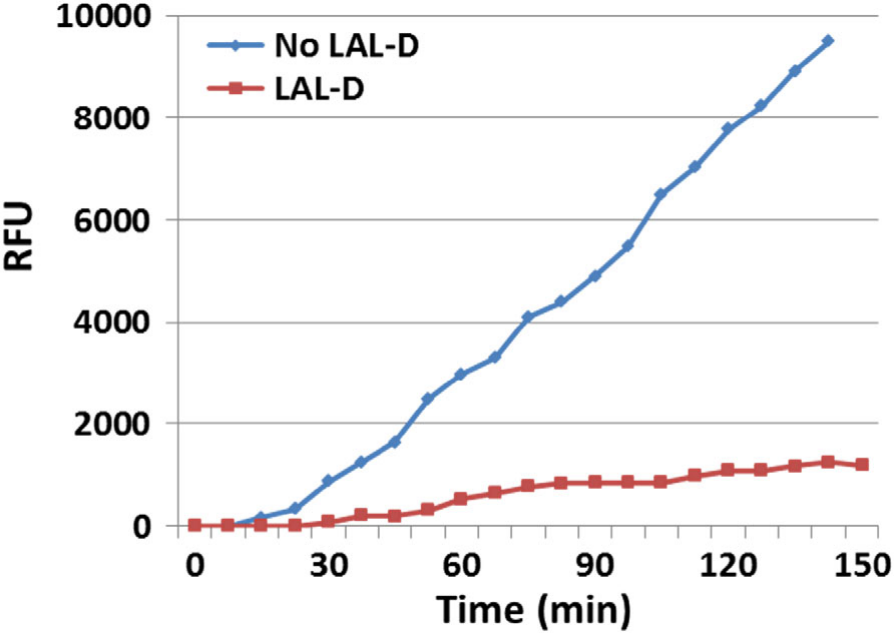
Typical kinetic curves of total lipase activity in DBS from an individual with no LAL‐D and a LAL‐D patient by the kinetic assay of 4‐MU‐Palm cleavage. DBS, dry blood spots; LAL‐D, lysosomal acid lipase deficiency; RFU, relative fluorescence units

Overall, the above data suggested that the 4‐MU‐Palm‐based assay itself, without Lalistat‐2, could be reasonably accurate in discriminating the nonaffected population from LAL‐D patients, despite a lack of absolute specificity. As a result, we elaborated on the development of a Lalistat‐2‐independent assay for the discrimination of LAL‐D patients using a kinetic modification of the Hamilton LAL assay. In the modified method, the release of 4‐MU from 4‐MU‐Palm (ie, total lipase activity) was measured in 5‐minutes intervals for 1 hour without the addition of Lalistat‐2. The rate of 4‐MU release was clearly higher in nonLAL‐D specimens than that in LAL‐D specimens (Figure 1). No 4‐MU‐Palm cleavage was registered during an incubation time of up to 150 minutes without the enzyme source (ie, without specimens; data not shown).

Next, we examined a set of the archived specimens submitted for LAL measurement using the kinetic assay of total lipase activity. The specimen set included: (1) 90 dB randomly selected out of 507 nonLAL‐D specimens with LAL activity > 0.07 nmol 4‐MU/punch/1 hour, (2) all available nonLAL‐D specimens with LAL activity < 0.07 nmol 4‐MU/punch/1 hour (15/23), and (3) DBS from 15 LAL‐D patients. As shown in Table 2, TL‐AUC_1h_ in nonLAL‐D specimens with LAL activity > 0.07 nmol 4‐MU/punch/1 hour was significantly higher than that in LAL‐D specimens (*P* < .001); the values of TL‐AUC_1h_ did not intersect between the two groups. A lower limit boundary for TL‐AUC_1h_, 2652 RFU*h/punch, was defined as 28% of the median TL‐AUC_1h_ found in nonLAL‐D specimens with LAL > 0.07 nmol 4‐MU/punch/1 hour, analogous to the lower limit for LAL corresponding to 28% of the median LAL activity in healthy individuals (see above). The examined 15 LAL‐D patients had TL‐AUC_1h_ values below this threshold (Tables 2 and 3). Moreover, in 13 out of 15 nonLAL‐D specimens with LAL < 0.07 nmol 4‐MU/punch/1 hour, TL‐AUC_1h_ was >2652 RFU*h/punch, being clearly separated from LAL‐D patients (Table 2). Thus, the kinetic total lipase activity assay was highly sensitive (100%) and specific (98%) with respect to detecting LAL‐D patients.

**Table 2:** Total lipase activity in DBS from individuals without LAL‐D and LAL‐D patients by the kinetic assay of 4‐MU‐Palm cleavage and expressed as TL‐AUC_1h_

Group (No.)	TL‐AUC_1h_, RFU*h/punch		No. (%) of specimens with TL‐AUC_1h_ < 2652 RFU*h/punch^a^
	Median (P25; P75)	Range	
No LAL‐D, LAL >0.07 nmol 4‐MU/ punch/1 h (90)	9471 (7048; 11 916)	4015‐23 585	0
No LAL‐D, LAL <0.07 nmol 4‐MU/punch/1 h (15)	4934 (3501; 5843)	2284‐9768	2 (13%)
LAL‐D (15)	1870 (1521; 2148)	357‐2608	15 (100%)

Abbreviations: LAL, lysosomal acid lipase; LAL‐D, lysosomal acid lipase deficiency; RFU, relative fluorescence units; TL‐AUC_1h_, area under the kinetic curve after 1 hour.

a The lower reference boundary for TL‐AUC_1h_ is defined as 28% of the normal median TL‐AUC_1h_.

**Table 3:** Patient characteristics

Patient (Gender)	*LIPA*		Age at diagnosis, years	Age at last report, years	LAL, nmol 4‐MU/punch/1 h	TL‐AUC_1h_, RFU*h/punch
	allele 1	allele 2				
LAL‐D_1 (F)	c.894G>A	c.796G>T	5	11	0.009	2023
LAL‐D_2 (F)	c.894G>A	c.894G>A	5	10	0.008	1720
LAL‐D_3 (M)	c.894G>A	c.796G>T	5	10	0.007	1520
LAL‐D_4 (M)^a^	c.894G>A	c.796G>T	5	9	NT	NT
LAL‐D_5 (F)	c.894G>A	c.894G>A	9	11	<0.001	1768
LAL‐D_6 (M)	c.398C>A	c.398C>A	6	8	0.050	1917
LAL‐D_7 (M)^b^	c.894G>A	**c.420G>A** c	5	8	0.010	2608
LAL‐D_8 (F)^b^	c.894G>A	**c.420G>A** c	9	12	0.020	2197
LAL‐D_9 (M)^a^	c.894G>A	**c.421del** d	14	16	0.052	2147
LAL‐D_10 (M)^a^	c.894G>A	c.894G>A	13	15	<0.001	NT
LAL‐D_11 (F)	c.894G>A	**c.911_912delinsT** e	5	7	<0.001	1869
LAL‐D_12 (M)^a^	c.894G>A	**c.420G>A** c	10	11	0.060	NT
LAL‐D_13 (F)	c.894G>A	**c.‐1‐2A>G** f	5	6	0.020	1046
LAL‐D_14 (F)	c.894G>A	c.894G>A	10	11	0.030	2458
LAL‐D_15 (F)^a^	c.894G>A	c.796G>T	6	9	0.001	1796
LAL‐D_16 (M)	c.894G>A	c.894G>A	33	33	0.010	1329
LAL‐D_17 (M)^a^	c.894G>A	c.894G>A	7	7	0.018	2118
LAL‐D_18 (F)^a^^,^ g	**c.442del** h	c.817_818del	3 months	4 months	0.010	357

*Note*. Five novel mutations detected in this study are indicated by bold typeface.

Abbreviations: F, female; LAL, lysosomal acid lipase; LAL‐D, lysosomal acid lipase deficiency; M, male; NT, Not tested; RFU, relative fluorescence units; TL‐AUC_1h_, area under the kinetic curve after 1 hour.

a Revealed during this study.

b Sibling patients.

c Nonsense, exon 4; stop gained, p.W140*; not present in the HGMD but described in Russia.^13^

d Deletion, exon 4; frameshift, p.Ala141Leufs*20.

e Insertion/deletion, exon 9; frameshift, p.Lys304Ilefs*26.

f Splicing, intron 1; affects the exon‐intron boundaries, disturbing the acceptor splice site of exon 2.

g Infantile‐onset LAL‐D (Wolman disease); deceased 1 month after diagnosis.

h Deletion, exon 5; frameshift, p.Ala148Glnfs*13.

To evaluate the precision of the kinetic assay, we calculated the interday variation (CV_i_) from repeated analyses of DBS with normal and low TL‐AUC_1h_ over a period of three days. Importantly, the kinetic assay exhibited low TL‐AUC_1h_ CV_i_ values both in normal and LAL‐D specimens (0.8% and 4%, respectively). In comparison, the Lalistat‐2‐dependent LAL assay in our hands was less precise, demonstrating CV_i_ values for normal and patient LAL activities of 18% and 109%, respectively.

### 3.4 LAL‐D patient characteristics

In this report, we included six new genetically confirmed LAL‐D cases detected during the present study as well as 12 retrospective patients who had been diagnosed with LAL‐D starting in 2012 at our institutions. Only limited patient characteristics were available, which are included in Table 3. Eight individuals were diagnosed at the age of ≤ 5 years, including one 3‐month‐old girl with a Wolman phenotype, who was deceased at the age of 4 months. In this patient group, LAL activity ranged from < 0.001 to 0.060 nmol 4‐MU/punch/1 hour, with a median of 0.010 nmol 4‐MU/punch/1 hour. The relative LAL activity varied from < 1% to 24% of the median found in healthy individuals. All patients, except patient LAL‐D_18, were alive at the time of publication.

*LIPA* analysis revealed nine different types of pathogenic alleles, including four novel variants according to the HGMD (Human Gene Mutation Database professional) and a recent review^2^ and one mutation (c.420G>A), which has been described in Russia in a conference paper,^13^ but is not present in the HGMD (Table 3). The most common variant was c.894G>A, which was found in 16 (89%) patients; six affected individuals were homozygous for c.894G>A. Novel disease‐causing variants are described in the footnote of Table 3. These included two small frameshift deletions in exons 4 and 5, one frameshift insertion/deletion in exon 9 and one nonsense mutation in exon 4. The remaining intronic mutation affected the exon‐intron boundaries, disturbing the acceptor splice site of exon 2.

## 4 DISCUSSION

The objectives of this study were 2‐fold. First, we analyzed the results of LAL activity measurements in a large cohort of individuals with suspected LAL‐D and demonstrated the sufficient accuracy of the Lalistat‐2‐independent kinetic modification of the total lipase activity assay in detecting LAL‐D affected individuals. Second, we provided molecular genetic data of 18 LAL‐D patients, including six individuals diagnosed during this study. To the best of our knowledge, this is the first extensive report describing LAL‐D patients from Russia.

In general, the Lalistat‐2‐dependent LAL assay demonstrated good analytical performance, exhibiting high sensitivity and specificity. However, this assay incorporates two enzymatic reactions, which unavoidably increase total assay error, as has been discussed elsewhere.^11^ The use of Lalistat‐2 was substantiated by a potential interference of nonLAL lipases while measuring LAL activity using 4‐MU‐Palm, which is not fully specific for LAL.^6^ Indeed, the proportion of Lalistat‐2‐independent lipase activity toward 4‐MU‐Palm in nonLAL‐D material has been estimated as 22%.^11^ In the present study, the median nonLAL lipase‐4‐MU‐Palm cleavage activity values in healthy individuals and LAL‐D patients were 38% and >99% of the total lipase activity, respectively. However, in our hands, the results of the enzymatic reaction without Lalistat‐2, that is, the total lipase activity measurement clearly separated healthy individuals and LAL‐D patients during the implementation study. To secure the discriminating power, instead of a single end‐point value, we recorded a series of the total lipase activity measurements during a kinetic assay. This approach allowed the development of a reliable index of the total lipase activity, TL‐AUC_1h_, calculated in a single enzymatic kinetic reaction, without adding Lalistat‐2. Given that there is no need for Lalistat‐2, the existence of two parallel enzymatic reactions and a reduced assay time in conjunction with high sensitivity, specificity, and reproducibility, the kinetic assay seems to be practical for LAL‐D screening.

In 4.3% of the examined DBS, we observed a low LAL activity without detectable *LIPA* alterations. A reduced enzymatic activity in DBS is usually ascribed to poor specimen quality.^14^, ^15^ DBS with gross preparation errors, such as clotting, disturbed integrity, low volume, and microbiological contamination, can be detected by visual inspection and excluded from analysis. However, obscure DBS failures, such as hematocrit or blood cell count variations, are difficult to assess.^15^ Reduced LAL activity was documented in individuals with advanced liver disease, including cryptogenic cirrhosis and cirrhosis of known etiology, who had lower WBC and PLT counts than control subjects.^10^ Moreover, a direct correlation between WBC and PLT counts and LAL activity in DBS from healthy individuals has been described.^16^ Thus, DBS‐based enzymatic assays, being extremely practical for screening with a defined threshold of known sensitivity and specificity, seem to be less feasible for precise quantitative studies, where preparations standardized for, for example, protein concentration or cell number, may be preferable.

Reportedly, 98 disease‐causing variants in *LIPA* have been identified to this point.^2^ Genetic analysis of 18 LAL‐D patients in our cohort disclosed nine pathogenic variants of *LIPA* alteration. Almost 90% of the patients carried the c.894G>A mutation, which is the most frequently reported *LIPA* allele in LAL‐D affected individuals.^1^, ^2^ The remaining disease‐causing mutations included small deletions and splicing errors as well as missense and nonsense mutations.

In conclusion, the described Lalistat‐2‐independent assay seems to represent a practical and accurate approach for investigating LAL activity within the LAL‐D diagnostic workflow.

## AUTHOR CONTRIBUTIONS

N.M. designed the study, supervised the laboratory work, analyzed the data and wrote the manuscript. E.B., T.S., N.V., A.S., O.G., and K.S. contributed to the conception and design of the study. E.B. performed enzymatic assays. A.P. and K.S. carried out genetic examination. T.S., N.V., A.S., and O.G. conducted clinical diagnostics and patient characterization. All authors contributed to data analysis, data interpretation, and reviewed drafts of the manuscript and approved the final draft for submission.

## CONFLICT OF INTERESTS

Nikolay Mayanskiy, Alexander Pushkov, Tatiana Strokova, Nikolay Vlasov, Andrej Surkov, Olga Gundobina have received a speaker honorarium from Alexion Pharma LLC, Russia. Ekaterina Brzhozovskaya and Kirill Savostianov declare that they have no conflict of interest.

## ETHICAL AND PATIENT CONSENT STATEMENTS

All procedures followed were in accordance with the ethical standards of the responsible committee on human experimentation (institutional and national) and with the Helsinki Declaration of 1975, as revised in 2000. Informed consent was obtained from all participants or their legally authorized representatives for being included in the study.
